# Soluble CD127 potentiates IL‐7 activity in vivo in healthy mice

**DOI:** 10.1002/iid3.530

**Published:** 2021-09-15

**Authors:** Nawaf A. Aloufi, Alaa K. Ali, Stephanie C. Burke Schinkel, Bengisu Molyer, Priscila O. Barros, Joanne E. McBane, Seung‐Hwan Lee, Jonathan B. Angel

**Affiliations:** ^1^ Department of Biochemistry, Microbiology and Immunology University of Ottawa Ottawa Ontario Canada; ^2^ King Faisal Specialist Hospital and Research Center Riyadh Saudi Arabia; ^3^ Chronic Diseases Program Ottawa Hospital Research Institute Ottawa Ontario Canada; ^4^ Canadian Institutes of Health Research (CIHR) Canadian HIV Trials Network (CTN) Vancouver British Columbia Canada; ^5^ Centre for Infection, Immunity and Inflammation University of Ottawa Ottawa Ontario Canada; ^6^ Department of Medicine, Division of Infectious Diseases, The Ottawa Hospital University of Ottawa Ottawa Ontario Canada

**Keywords:** CD4^+^ T‐cell, CD8^+^ T‐cell, interleukin‐7 (IL‐7), soluble IL‐7Rα (sCD127), T‐cell proliferation

## Abstract

**Introduction:**

Soluble forms of cytokine receptors can be involved in the endogenous regulation of cytokine activity. Soluble interleukin 7 receptor α (sCD127) naturally binds IL‐7, therefore there is interest in its potential application as an immunotherapeutic agent to regulate IL‐7. With the hypothesis that sCD127 enhances IL‐7 activity, thus promoting T‐cell proliferation in vivo, we sought to assess the effect of sCD127, IL‐7 or IL‐7 + sCD127 treatment on CD4^+^ and CD8^+^ T‐cells in the blood and spleen of mice.

**Methods:**

Peripheral blood mononuclear cells and splenocytes were prepared, and analyzed for T‐cell number, phenotype and proliferation (Ki67^+^) by flow cytometry.

**Results:**

IL‐7 treatment induced T‐cell proliferation, increased T‐cell number, and triggered T‐cell differentiation each of which was enhanced with the addition of sCD127. IL‐7 + sCD127 treatment significantly increased spleen weight over that seen with IL‐7 treatment alone. More pronounced proliferation and a greater increase in cell number was observed in CD8^+^ T‐cells relative to the effect on CD4^+^ T‐cells.

**Conclusions:**

These findings suggest that the addition of sCD127 enhances IL‐7‐mediated T‐cell proliferation and suggests a potential therapeutic use for sCD127.

## Graphical abstract

IL‐7 alone can have a positive effect on T‐cell proliferation and T‐cell differentiation from a naive T‐cell. In this mouse study, we found that complexing IL‐7 with soluble CD127 could enhance these positive effects, suggesting a therapeutic use in vivo.

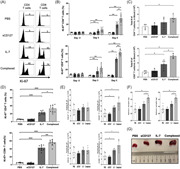

## 1 INTRODUCTION

Interleukin 7 (IL‐7) is a hematopoietic growth factor that promotes murine B cell precursor growth, and a master regulator of T‐cell development and homeostasis.^1^, ^2^ IL‐7 signals are transduced by IL‐7 receptor (IL‐7R), a heterodimer complex composed of IL‐7R‐α chain (CD127) and the common gamma‐chain (CD132/IL‐2Rγ/γc). CD127 is also part of the thymic stromal lymphopoietin receptor. IL‐7R is expressed on cells of lymphoid origin, including developing and mature T‐cells, and nonimmune cells, including bone marrow stromal cells.^3^

Some soluble cytokine receptors participate in cytokine regulation by exerting antagonistic effects and acting as natural competitors of the membrane receptor,^4^ while others enhance cytokine signals by preventing cytokine clearance. Soluble IL‐2Rα binding to IL‐2 can reduce its ability to signal, inhibiting IL‐2‐dependent T‐cell activation and proliferation.^5^ A clinically relevant example is that of soluble TNFR2‐Fc (Etanercept) which by blocking TNF‐α activity has become a frequently prescribed therapeutic agent for rheumatoid arthritis, psoriasis and other autoimmune diseases.^6^, ^7^ Typically, the higher the affinity of the soluble receptor for the ligand, the more potent the inhibition of the signal; however, some studies suggest that sIL‐4R and sIL‐15R enhance ligand activity by increasing cytokine stability.^8^, ^9^

CD127 exists in membrane‐bound and soluble forms (sCD127).^10^ sCD127 generally circulates at a molar excess relative to IL‐7, suggesting a role for sCD127 in controlling IL‐7 activity, but the mechanisms by which this occurs in vivo are unknown.^11^ Excessive sCD127 has been linked to autoimmune disease.^12^ In experimental autoimmune encephalomyelitis (EAE), a murine model of MS, administration of sCD127 with IL‐7 increased the bioactivity of IL‐7 and was associated with increased disease severity.^13^

The importance of delineating the role of sCD127 in mediating IL‐7 activities arises from the fact that in healthy individuals, sCD127 has been found to circulate at concentrations several log higher than that of IL‐7 (nmol/ml compared with pmol/ml levels).^11^ In addition, various degrees of binding affinity of sCD127 to IL‐7 have been demonstrated. Specifically, when sCD127 is in a homodimer it binds with moderate‐affinity to IL‐7, whereas the heterodimeric form sCD127/soluble common gamma chain receptor (sCD132) has high binding affinity to IL‐7.^13^ The process by which sCD127 modulates IL‐7 responses in vivo have not been well‐elucidated and both attenuating and promoting effects have been described in vitro. sCD127 has been reported to block IL‐7 signaling as well as inhibit STAT5 phosphorylation and IL‐7‐mediated proliferation.^14^, ^15^ However, other studies have demonstrated that sCD127 acts as a carrier protein for IL‐7, thereby increasing the half‐life of IL‐7 and promoting the biological activity of IL‐7.^13^ Furthermore, a recent study in our laboratory has reported that preincubation of a chimeric IL‐7Rα‐Fc receptor (an artificial protein that mimics natural sCD127, consisting of CD127 extracellular domain and human Fc‐fragment) with IL‐7 enhanced IL‐7‐mediated human and murine T‐cell proliferation in vitro.^16^ Such observations pave the way for subsequent work to establish the role of sCD127 *in vivo*, the main focus of the present study. This is particularly relevant as there are currently 15 clinical trials registered worldwide evaluating the ability of IL‐7 to enhance immune reconstitution.^17^ Here, we evaluate IL‐7 treatment on T‐cell proliferation in healthy mice, and hypothesize that sCD127 will enhance IL‐7 activity.

## 2 MATERIALS AND METHODS

### 2.1 Mice

C57BL/6 mice aged 7–12 weeks and gender‐matched for experiments. The mice were kept under a specific pathogen‐free condition with microisolator cages housed in the University of Ottawa's main rodent facility. Experiments were approved by the University of Ottawa Animal Ethics Committee and the Canadian Council on Animal Care.

### 2.2 Recombinant murine IL‐7 and sCD127 treatment of healthy mice

**Figure 1:**
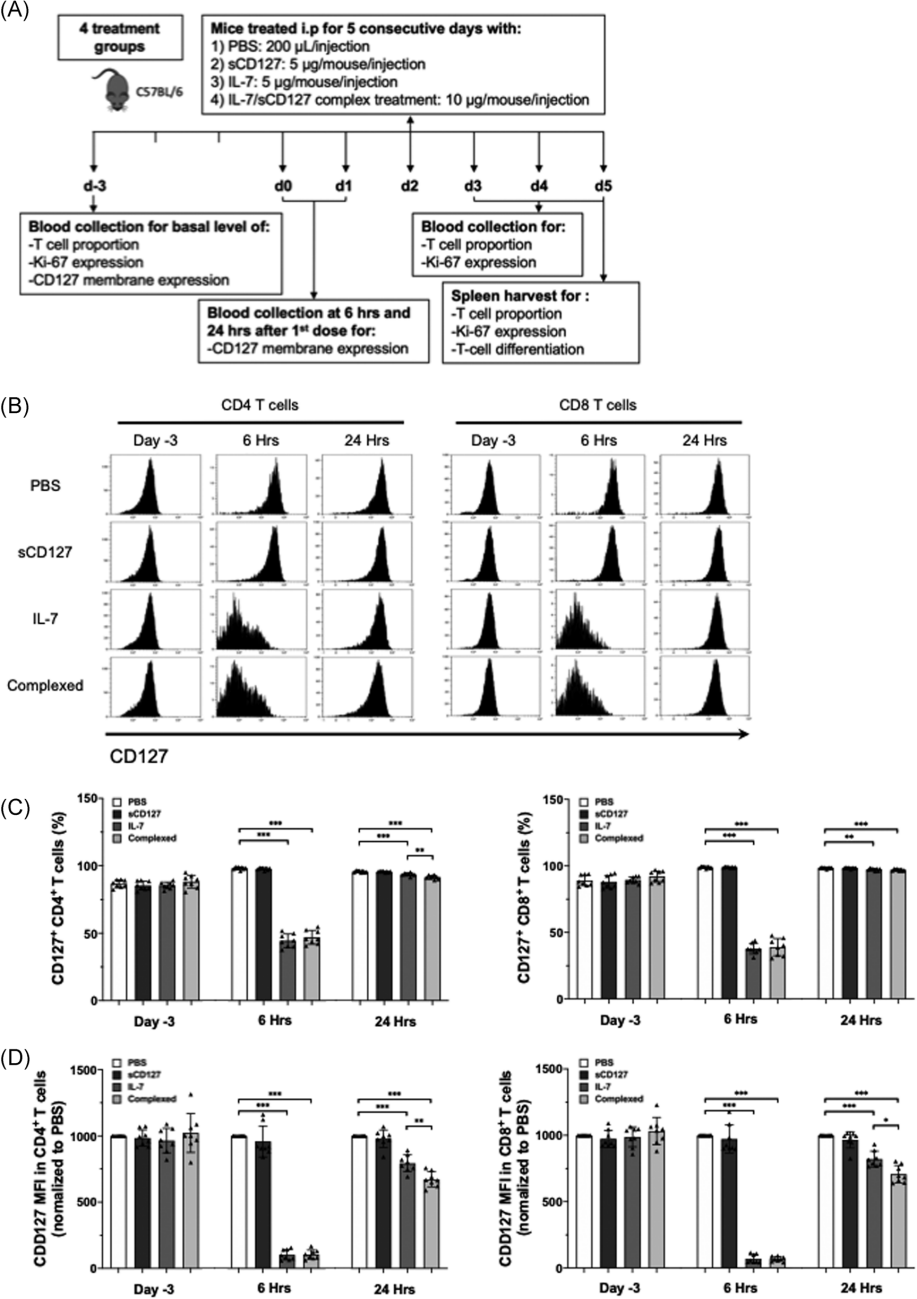
The effect of IL‐7 and sCD127 on CD127 expression on CD4^+^ and CD8^+^ T‐cells in healthy C57BL/6 mice. (A) Experimental timeline of healthy age‐matched female C57BL/6 mice. Mice were divided into four groups, and treated for 5 consecutive days either with sCD127 alone, IL‐7 alone, a combined dose of sCD127 and IL‐7 (preincubated at 37°C for 30 min), or PBS. (B) Flow cytometric analysis of CD127 expression on CD4^+^ and CD8^+^ T‐cells. (C) The proportion of CD4^+^ T‐cells expressing CD127 (C, left), and CD8^+^ T‐cells expressing CD127 (C, right) in the blood before treatment (Day 3), 6 h posttreatment (6 Hrs), and 24 h posttreatment (24 Hrs). (D) CD127 MFI in CD4^+^ T‐cells (D, left) and CD8^+^ T‐cells (D, right) normalized to CD127 MFI of a PBS‐treated group. Data are pooled from two independent experiments, *n* = 8 mice per group. Data represent mean ± *SD* and statistical analysis was completed using a one‐way ANOVA for more than two groups, and an unpaired nonparametric Mann–Whitney test was used for comparisons between the two groups. *p* Values are listed as either not significant (ns, *p* > .05) or significant, with *p* values grouped as follows: *p < .05, **p < .01, ***p < .001, or ****p < .0001. An asterisk above a column bar represents a statistically significant difference between the indicated treatment and PBS‐treated mice at the same time point. ANOVA, analysis of variance; PBS, phosphate‐buffered saline

For mouse treatments, recombinant murine IL‐7 (catalog #: 217‐17; endotoxin level: <0.01 ng/mg cytokine as determined by the LAL assay) was purchased from PeproTech, and IL‐7Rα/CD127 Fc Chimera (catalog #: 747‐MR‐050; endotoxin level: <1.0 Endotoxin Units/mg of protein by LAL method) was purchased from R&D Systems. Mice were injected intraperitoneally for 5 consecutive days with 200 μl phosphate‐buffered saline (PBS); 5 μg of IL‐7; 5 μg of sCD127; or a 10 μg of IL‐7 + sCD127 (5 μg each) (Figure 1A). All treatments were diluted in sterile PBS. To allow the complex to form, 5 μg of IL‐7 was mixed with 5 μg of sCD127 and incubated for 30 min (mins) at 37°C immediately before administering the treatment (protocol adapted from previous reports^16^, ^18^).

### 2.3 Cell isolation

Blood was collected in heparin‐coated capillary tubes from the saphenous vein of mice at indicated time points. Approximately 40 μl of blood was collected in heparin‐coated capillary tubes (BD Vacutainer, cat# 026896) via a saphenous vein of experimental mice at appropriate time points. The blood samples were kept on ice throughout the procedures. After collection, blood was then mixed in 1 ml of PBS in a 15 ml conical centrifuge tube (cat# 339650; Thermo Scientific), and washed by centrifugation for 5 min at 4°C and at 652*g*. The supernatant was discarded, and the red blood cells (RBCs) were then lysed in 1 ml Ammonium–Chloride–Potassium (ACK) lysis buffer, pH 7.4 for 1 min. Then, 9 ml of RPMI‐1640 was added immediately, filtered through a 200 μm nylon mesh (cat# 0320045; Elko Filtering) cell strainer and washed by centrifugation once for 5 min at 4°C and at 652*g*. The cells were then resuspended in 2 ml media, counted on a hemocytometer, washed by centrifugation again for 5 min at 4°C and at 652*g*, and then resuspended in staining buffer (SB) for further analysis. A 40 μl sample yields ~1–2 × 10^5^ peripheral mononuclear cells (PBMCs) with ~40% T‐cells.

Spleens were harvested from mice at endpoints as indicated. Spleens were gently dissociated in media and pushed through a 70 μm nylon cell strainer. Splenocytes were then lysed in ACK lysis buffer, pH 7.4, washed by centrifugation, and counted. This yields 5–10 × 10^7^ total leukocytes per spleen, and ~40% of the splenic leukocytes are T‐cells. Splenocytes were resuspended in at assay‐specific cell densities.

### 2.4 Flow cytometric analysis

Cell suspensions were prepared from mouse (blood or spleen) in PBS containing 2% fetal bovine serum (FACS buffer) at a density of 1 × 10^6^ cells/well. Cells were subsequently washed with FACS buffer and stained with a cocktail of fluorochrome‐conjugated monoclonal antibodies specific for CD3 (145‐2C11), CD4 (RM4‐5), CD8α (53‐6.7), Ki‐67 (B56), CD44 (IM7) (BD Biosciences), CD127 (SB/199), and CD62L (MEL‐14) (eBioscience). Fixable Far‐Red Live/Dead was added (Invitrogen) at 4°C for 25 min, washed, and fixed in 2% paraformaldehyde. The phenotype of the cells was determined using the following fluorescent monoclonal antibodies against cell surface or viability markers including anti‐CD3, anti‐CD4, and anti‐CD8α for T‐cells; anti‐NK1.1 for NK and NKT cells; fixable viability stain for dead cells; anti‐CD127 for membrane surface expression and mean fluorescence intensity of CD127; and anti‐CD62L and anti‐CD44 for T‐cell subset identification. More information about staining panels and controls used to examine the cells can be found in the Supporting Information (Table S1). For Annexin V staining, the cells were washed twice with Annexin V binding buffer (556454; BD Biosciences), then stained with 1 μl PE‐Annexin V antibody (556422; BD Biosciences) in 50 μl binding buffer for 15 min at RT. 100 μl binding buffer was added, mixed, then the samples were analyzed immediately on a BD Fortessa. For intracellular staining of Ki‐67, after surface staining was completed, the cells were fixed and permeabilized using Foxp3 Transcription Factor Staining Buffer Set (cat#00552300; eBioscience), and was carried out following the manufacturer's protocol. Briefly, to fix the cells 1 part of Foxp3—Fixation Concentrate (cat# 00512343) was diluted into three parts of the Fixation/Permeabilization Diluent (cat# 00522356). The cells were handled for the subsequent steps at room temperature (RT). Briefly, cells were permeabilized twice by adding 150 μl of perm buffer and washed once by centrifugation at 453*g* for 4 min at RT. For permeabilization, 1× permeabilization buffer was made by diluting 10X Permeabilization concentrate (cat# 00833356) with deionized water. The cells were then stained with V450‐conjugated antimouse monoclonal Ki‐67 antibodies (0.2 μl/10^6^ cells) in 40 μl of perm buffer and incubated at RT for 30 min in the dark. The cells were washed twice with perm buffer and resuspended in 300 μl of SB for further flow cytometric analysis. Relative fluorescence intensities were measured with LSRFortessa™ or FACSCelesta™ (BD Biosciences). Data were analyzed using Kaluza software v2.0 (Beckman Coulter).

### 2.5 Statistical analysis

All statistical analyses were determined using GraphPad Prism 9.0 software. Statistical significance was calculated by performing a one‐way analysis of variance (ANOVA) followed by an unpaired nonparametric Mann‐Whitney test. Values with a *p* value <.05 were considered significant. Data are presented as mean ± standard deviation (*SD*), and *p* values are represented as not significant (ns), *p < .05, **p < .01, ***p < .001, and ****p < .0001.

## 3 RESULTS AND DISCUSSION

### 3.1 Soluble IL‐7Rα‐Fc does not inhibit IL‐7 induced downregulation of surface CD127 expression on T‐cells

IL‐7 plays a nonredundant role in T‐cell homeostasis, providing T‐cells with the survival and proliferative signals needed to maintain a constant level in the periphery.^19^ Under states of equilibrium, T‐cells are tightly controlled by various homeostatic mechanisms. These require IL‐7 signaling and other essential factors including the interaction of TCR on T‐cells with self‐peptides on APCs and other cytokine signals.^20^ Here, we induced T‐cell proliferation in healthy mice by administering exogenous IL‐7 and evaluated the effect of adding sCD127. To our knowledge, this is the first study to report the effect of sCD127 administration in healthy mice. In the current study, we adopted the approach as published by Andersson et al 2011,^18^ in which it was demonstrated that administering a 5 μg/dose of either IL‐7 alone or in a combination with an equal dose of sCD127 increased T‐cell frequency. Lower doses of IL‐7 + /‐sCD127 did not induce such an effect in our model system (data not shown). Once IL‐7 binds to its target cells, it downregulates surface IL‐7R as part of a negative feedback mechanism.^21^ PBMCs were isolated from healthy mice 6 and 24 h following treatment initiation and surface expression of CD127 on CD4^+^ and CD8^+^ T‐cells was examined by flow cytometry using the indicated antibodies. At baseline, 85% of each CD4^+^ and CD8^+^ T‐cells expressed CD127. Six hours posttreatment with IL‐7 or IL‐7 + sCD127, 50% of CD4^+^ T‐cells expressed CD127 (Figure 1C, left), while 40% of CD8^+^ T‐cells expressed CD127 (Figure 1C, right). sCD127 treatment alone did not alter CD127 expression (Figure 1C). Interestingly, CD127 downregulation was transient and CD127 expression returned to basal levels 24 h posttreatment. Precomplexing sCD127 with IL‐7 did not alter the IL‐7‐induced effect and, importantly, inhibition of the IL‐7‐mediated downregulation of the membrane‐bound CD127 was not observed. This contrasts a previous in vitro report where sCD127 inhibited IL‐7 signal transduction and the phosphorylation of STAT5 and AKT, two downstream molecules in IL‐7 signaling.^14^

We also investigated the levels of CD127 expression on the surface of CD4^+^ and CD8^+^ T‐cells and observed that IL‐7 with or without sCD127 significantly reduced CD127 MFI at 6 h posttreatment (Figure 1B,D). Notably, the level of CD127 expression remained low with both IL‐7 and IL‐7 + sCD127 treatment at 24 h despite the return to normal of the proportion of cells expressing CD127 (Figure 1C). Interestingly, combined treatment resulted in significantly lower level of CD127 in both CD4^+^ and CD8^+^ T‐cells compared with mice treated with IL‐7 alone suggesting a prolonged effect of IL‐7 when combined with sCD127 (Figure 1D). Next, to consider the possibility that a decrease in CD127 expressing cells was not a result of selective loss of these cells from the circulation, we investigated whether treatment with IL‐7 and/or sCD127 would altered T lymphocyte number in the blood. No change in the proportion of total CD4^+^ T‐cells was observed (Figure S1C, left), and while there was a significant decrease in CD8^+^ T‐cells seen at 6 h, this was modest and unlikely to explain the decrease in CD127 expression in the circulating cells (Figure S1C, right).

### 3.2 Murine soluble IL‐7RΑ‐Fc enhances IL‐7‐mediated T‐cell proliferation and cell counts in mice blood and spleen

**Figure 2:**
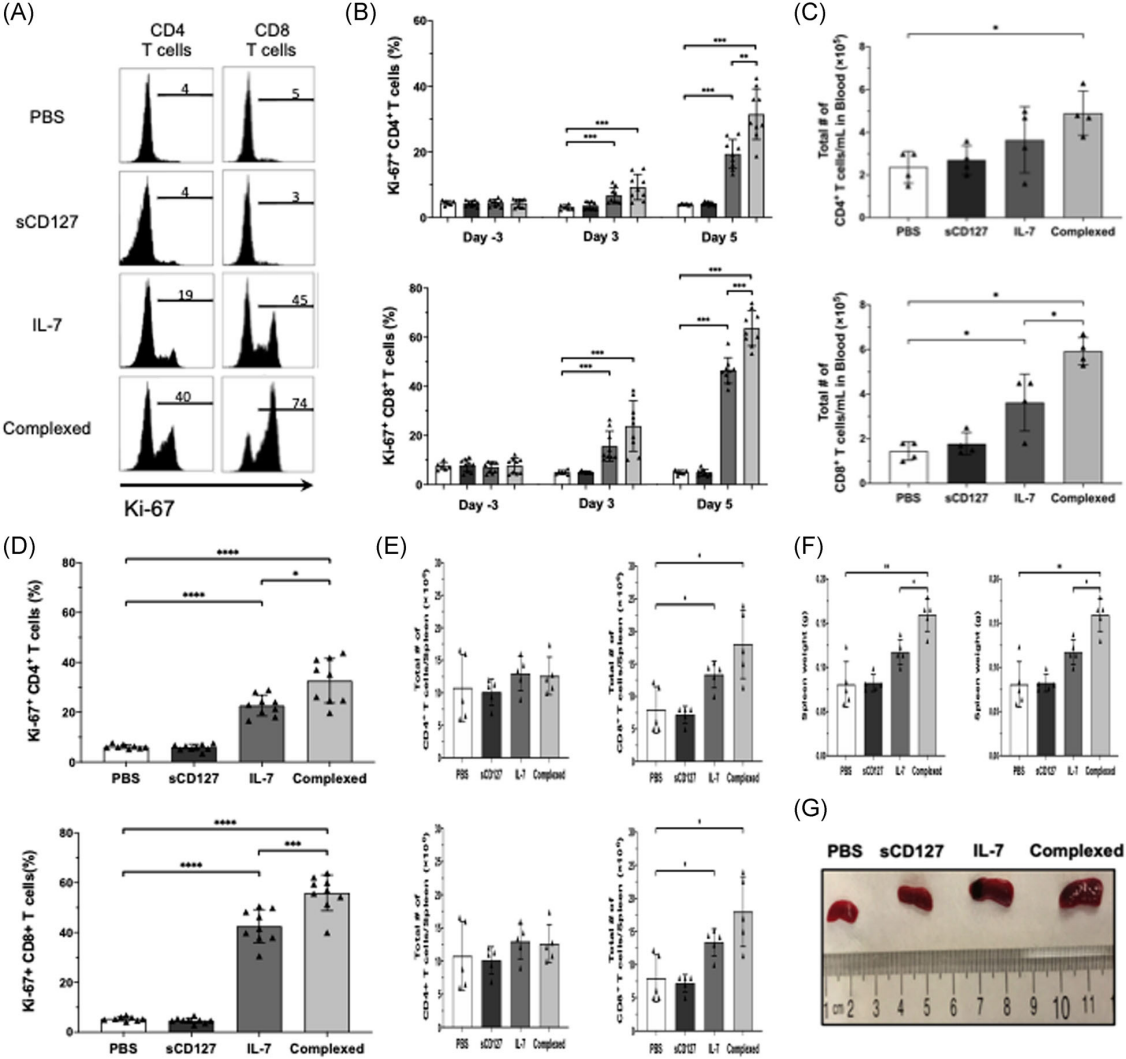
The effect of IL‐7 and sCD127 on T‐cell proliferation and T‐cell numbers in the blood and spleen of healthy C57BL/6 mice. Healthy C57BL/6 mice were divided into 4 groups, and treated by i.p. injection for 5 consecutive days with either: 5 μg of IL‐7, 5 μg of sCD127, 10 μg of both treatments complex (preincubated for 30 min at 37°C), or 200 μl of PBS alone (vehicle control). (A) Representative histograms representing Ki‐67 expression within blood CD4^+^ and CD8^+^ T‐cells at Day 5 posttreatment. (B) The frequency of Ki67^+^ cells among CD4^+^ T‐cells (B, top) and CD8^+^ T‐cells (B, bottom) are demonstrated. Data are pooled from two independent experiments, *n* = 9 mice per treatment group and *n* = 8 mice per PBS control group. (C) The total number of CD4^+^ T‐cells or CD8^+^ T‐cells was measured in the blood of mice at Day 5. The graphs show the total number of CD4^+^ T‐cells (C, top) or the total number of CD8^+^ T‐cells (C bottom). Mice were divided into four groups as indicated before, *n* = 4 mice per group. (D)–(G) The effect of five consecutive days of sCD127 and/or IL‐7 treatment on T‐cell proliferation in the spleen of healthy C57BL/6 mice was evaluated. On the day following the final i.p. Injection, mice were sacrificed, and the spleens were harvested. (D) The frequency of Ki‐67^+^ cells in CD4^+^ T‐cells (D, top) and CD8^+^ T‐cells (D, bottom) isolated from the spleen are shown. Data are pooled from two independent experiments, *n* = 9 mice per treatment group and *n* = 8 mice per PBS control group. (E) The total number of CD4^+^ T‐cells or CD8^+^ T‐cells was measured in the spleen of mice at Day 5. The graphs show the total number of CD4^+^ T‐cells (E, top), or the total number of CD8^+^ T‐cells (E, bottom). Mice were divided into four groups as indicated before, *n* = 5 mice per group. (F) Harvested spleens were weighed (*n* = 5 mice per group) and representative images of whole spleens from each treatment group are shown (G). All data represent mean ± *SD*. Statistical analysis was completed using a one‐way ANOVA for more than two groups, and an unpaired nonparametric Mann–Whitney test was used for comparisons between the two groups. Significant *p* values are listed as either *p < .05, **p < .01, ***p < .001, or ****p < .0001. ANOVA, analysis of variance; PBS, phosphate‐buffered saline

Consistent with one report in tumor bearing mice,^18^ we found that treating healthy mice with IL‐7 for 5 days enhanced proliferation of CD4^+^ and CD8^+^ T‐cells as measured by expression of the nuclear antigen Ki‐67, a marker of cellular proliferation. In the blood at basal state, CD4^+^ and CD8^+^ T‐cells were found to be ~5% and ~9% Ki67^+^, respectively. IL‐7 treatment significantly increased the percentage of Ki‐67^+^ CD4^+^ and CD8^+^ T‐cells. By Day 3 of IL‐7 treatment, 7% of CD4^+^ T‐cells and 16% of CD8^+^ T‐cells were Ki‐67^+^. By Day 5, 20% of CD4^+^ T‐cells and 45% of CD8^+^ T‐cells were Ki‐67^+^ (Figure 2B). Interestingly, IL‐7 + sCD127 further enhanced the effect on T‐cell proliferation. At Day 3, 10% and 25% of the circulating CD4^+^ T‐cells and CD8^+^ T‐cells were Ki‐67^+^, and at Day 5, 30% and 60% of the circulating CD4^+^ T‐cells and CD8^+^ T‐cells were Ki‐67^+^, respectively (Figure 2B). There was no difference in the percentage of cells expressing Ki‐67 when mice were treated with sCD127 alone compared with pretreatment levels or to those treated with PBS. The Ki‐67 increase was consistently greater in CD8^+^ T‐cells than in CD4^+^ T‐cells (Figure 2B).

Ki‐67^+^ expression was also assessed in the spleen of mice treated with PBS, sCD127, IL‐7 or IL7 + sCD127. Spleens harvested at Day 5 were stained for Ki‐67. IL‐7 treatment increased the expression of Ki‐67 in both CD4^+^ and CD8^+^ T‐cells. Following IL‐7 treatment, 22% of the CD4^+^ T‐cells and 40% of the CD8^+^ T‐cells in spleen were Ki‐67^+^ compared with 5% after PBS treatment (Figure 2D). sCD127 alone did not change the proportion of Ki‐67^+^ cells. Interestingly, a greater effect on T‐cell proliferation was observed in CD4^+^ and CD8^+^ T‐cells with IL‐7 + sCD127 compared with IL‐7 alone. Nearly 32% of splenic CD4^+^ T‐cells (vs. 22% with IL‐7 alone) and 56% of splenic CD8^+^ T‐cells (vs. 40% with IL‐7 alone) were positive for Ki‐67 following treatment with IL‐7 + sCD127. The results reported here (Figure 2B,D) are consistent with previous data demonstrating that IL‐7 + sCD127 has a synergistic effect on IL‐7 activity *in vitro* and in vivo.^13^, ^16^

How sCD127 potentiates IL‐7 activity remains unknown. Soluble cytokine receptors can enhance cytokine activity: (1) by acting as a carrier protein, increasing the half‐life of the cytokine and promoting the biological activity; or (2) via trans‐presenting the cytokine to cells that do not express the complete receptor.^8^, ^9^ It is proposed that in vivo sCD127 potentiates IL‐7 function by extending its half‐life, though more work is needed to preclude trans‐presentation.^13^

With the positive impacts of both IL‐7 and its combination with sCD127 seen on T‐cell proliferation in peripheral lymphocytes and in the spleen, we anticipated an increase in total lymphocyte numbers of treated mice as well. Healthy mice were treated as outlined, with total CD4^+^ and CD8^+^ T‐cells in blood determined by flow cytometry. IL‐7‐treated mice demonstrated a significant increase in CD8^+^ T‐cell count whereas the increase in CD4^+^ T‐cell count did not reach statistical significance with IL‐7 alone (Figure 2C). At Day 5, the total CD4^+^ T‐cell count in PBS‐treated mice was 2 × 10^5^ cells/ml of blood, while this increased to 4 × 10^5^ cells/ml in IL‐7‐treated mice (Figure 2C). Total CD8^+^ T‐cell count in PBS‐treated mice was 1.5 × 10^5^ cells/ml of blood, and increased to 4 × 10^5^ cells/ml in IL‐7‐treated mice (Figure 2C). Interestingly, the addition of sCD127 only augmented the effect observed by IL‐7 in CD8^+^ T‐cell, while in CD4^+^ T‐cells there was a slight but not statistically significant increase. The CD4^+^ T‐cell counts were 5 × 10^5^ cells/ml in IL‐7 + sCD127 versus 4 × 10^5^ cells/ml in the IL‐7 group, whereas CD8^+^ T‐cell counts were ~6 × 10^5^ T‐cells/ml in IL‐7 + sCD127 versus ~4 × 10^5^ T‐cells/ml in IL‐7 treated mice (Figure 2C).

The total number of CD4^+^ and CD8^+^ T‐cells expressing Ki‐67 in blood was also enumerated. At Day 5, there was a significant increase in the number of both Ki‐67 positive CD4^+^ and CD8^+^ T‐cells after IL‐7 treatment (Figure S2B). Similar to that seen with T‐cell number, the addition of sCD127 significantly augmented the effect of IL‐7 on the number of Ki‐67^+^CD4^+^ and CD8^+^ T‐cells in blood (Figure S2B).

In the spleen, there were ~11 × 10^6^ CD4^+^ cells/spleen in PBS‐treated mice compared with ~13 × 10^6^ cells/spleen in mice treated with IL‐7 or IL‐7 + sCD127 (Figure 2E). Total CD8^+^ T‐cell counts were significantly increased following IL‐7 and IL‐7 + sCD127 treatment compared with PBS‐treated mice. There were ~8 × 10^6^ cells/spleen in PBS‐treated mice compared with 13 × 10^6^ cells/spleen and 18 × 10^6^ cells/spleen in the IL‐7‐ and IL‐7 + sCD127‐treated mice, respectively (Figure 2E). There was no statistically significant difference in CD8^+^ T‐cell count between mice treated with IL‐7 versus IL‐7 + sCD127.

Additionally, we have also enumerated the number of both CD4^+^ and CD8^+^ T‐cells expressing Ki‐67 in spleen. There was a significant increase in the number of both Ki‐67^+^ CD4^+^ and CD8^+^ T‐cells when mice were treated with either IL‐7 alone or IL‐7 in combination with sCD127 compared with PBS‐treated mice (Figure S2C). The addition of sCD127, however, only augmented the effect observed by IL‐7 in CD8^+^ T‐cell, while in CD4^+^ T‐cells there was a slight but not statistically significant increase (Figure S2C). The increase in T‐cell proliferation and cell number observed in the spleen correlated with a significant enlargement in spleen size (Figure 2F,G). T‐cell proliferation and T‐cell numbers increased following IL‐7 treatment, which was enhanced with the addition of sCD127 with a greater increase in CD8^+^ T‐cell number compared with that of CD4^+^ T‐cells. These differences between CD4^+^ and CD8^+^ T‐cells are consistent with reports by Geiselhart et al. and Sportès et al.,^22^, ^23^ but the mechanisms responsible for these differences are unknown. There might be distinct underlying factors regulating the homeostatic proliferation of CD8^+^ and CD4^+^ T‐cells in response to IL‐7. It is also possible that supraphysiologic concentrations of IL‐7 negatively impact CD4^+^ T‐cell proliferation, potentially explaining the difference between the T‐cell subsets.^24^ Guimond et al. suggested that elevated IL‐7 levels downregulate MHC II expression on APCs, thus indirectly interfering in the TCR‐mediated homeostatic proliferation of CD4^+^ T‐cells.^25^ Future work may be needed to optimize conditions required to maximize CD4^+^ T‐cell proliferation. While the increased T‐cell number can likely be attributed to the expansion of naïve T‐cells in the periphery or through the redistribution of T‐cells in the secondary lymphoid organs, it is also possible that treatment with rIL‐7 + sCD127 may impact T‐cell homeostasis via altered thymic selection of T‐cell subsets. However, this is unlikely given that the process of thymic T‐cell maturation has been predicted to take 3–4 weeks.^26^, ^27^

### 3.3 Differential expansion of central memory CD8^+^ T‐cells and effector type CD4^+^ T‐cell subsets is augmented by combination of IL‐7 and sCD127 treatment

**Figure 3:**
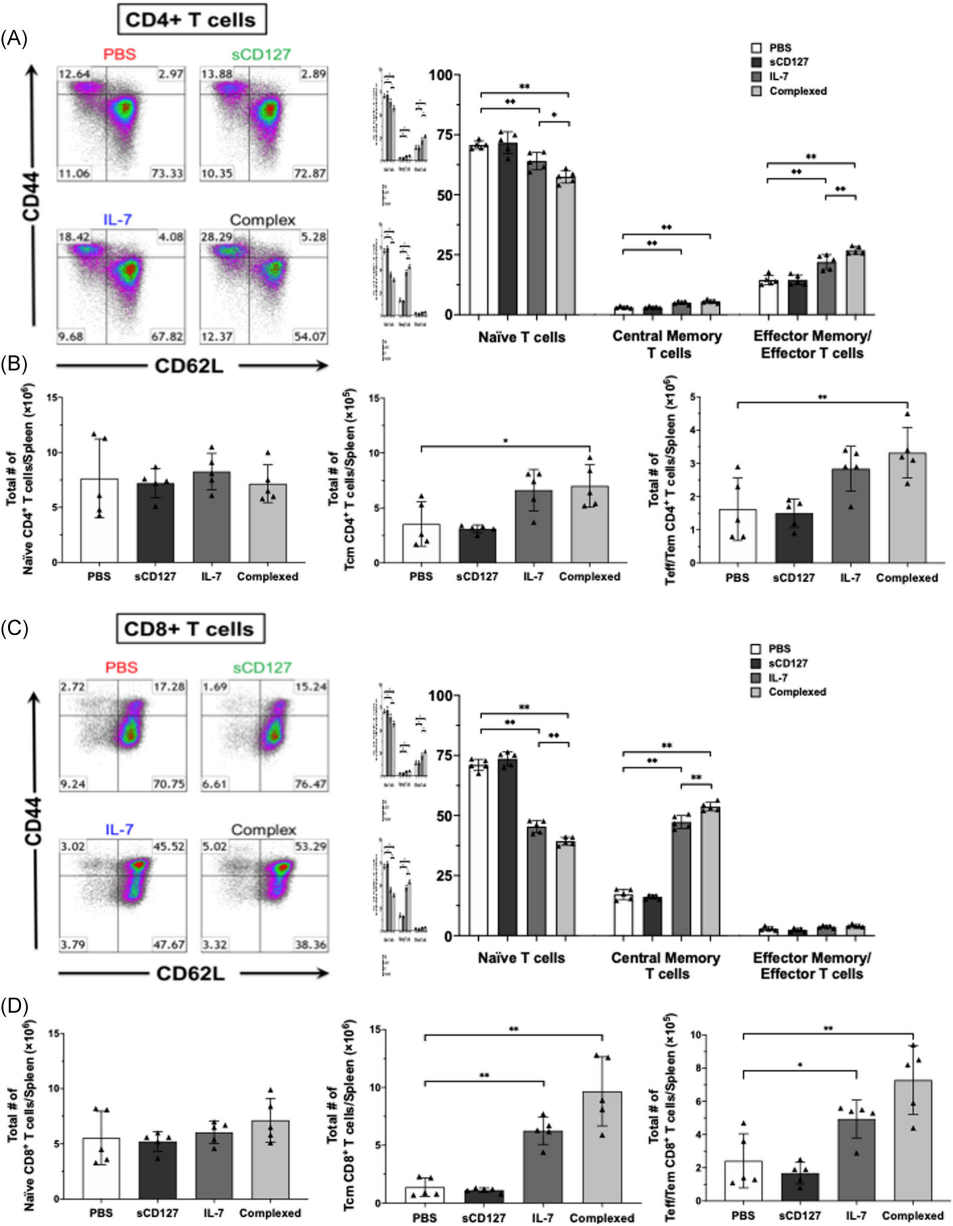
The effect of IL‐7 and sCD127 on spleen T‐cell subsets in healthy C57BL/6 mice. Mice were divided four groups and treated for 5 consecutive days (d0–d4) with either: 5 μg of IL‐7, 5 μg of sCD127, a complex 5 μg of IL‐7 and 5 μg of sCD127 (preincubated for 30 min at 37°C) or PBS alone. On the day following the final i.p. injection (d5), mice were sacrificed, and the spleens were harvested. (A, left panel) Representative scatter plots gated on CD4^+^ T‐cells showing expression levels of CD44 and CD62L for the four different treatment groups (A, right panel) Quantification of CD4^+^ T‐cells isolated from the spleen expressing either naïve T‐cell (CD44 low, CD62L high); central memory T‐cell (CD44_high_, CD62L_high_); or effector T‐cell (effector T‐cells or effector memory T‐cells) (CD44_high_, CD62L_low_) markers are shown. (B) The total number of naïve CD4^+^ T‐cells (B, left), central memory CD4^+^ T‐cells (B, center), and effector CD4^+^ T‐cells (B, right) are demonstrated. (C, left panel) Representative scatter plots gated on CD8^+^ T‐cells showing expression levels of CD44 and CD62L for the four different treatment groups. (C–right panel) Quantification of CD8^+^ T‐cells isolated from the spleen expressing either naïve T‐cell, central memory T‐cell, or effector T‐cell markers. (D) The total number of naïve CD8^+^ T‐cells (D, left), central memory CD8^+^ T‐cells (D, center), and effector CD8^+^ T‐cells (D, right) are demonstrated. Data represent mean ± *SD*. Statistical analysis was completed using a one‐way ANOVA for more than two groups, and an unpaired nonparametric Mann–Whitney test was used for comparisons between the two groups. Significant *p* values are listed as either *p < .05, **p < .01, ***p < .001, or ****p < .0001. ANOVA, analysis of variance; PBS, phosphate‐buffered saline

Experiments with healthy mice aimed to assess the roles of IL‐7 and sCD127 in different T‐cell subsets. These mice were housed in a pathogen‐free environment, hence, any phenotypic changes reported among CD4^+^ and CD8^+^ T‐cells can be attributed to the treatments. Splenic T‐cells were evaluated for naïve T‐cells (Tn), central memory T‐cells (Tcm), and effector T‐cells (Teff) and effector memory (Tem) T‐cells populations using antibodies against CD62L and CD44. Tn cells were defined as CD62L_high_ CD44_low_, Tcm as CD62L_high_ CD44_high_, and Teff & Tem T‐cells, both referred as effector T‐cells and defined as CD62L_low_ CD44_high_. After 5 days of treatment, T‐cell subsets from spleens were quantified. Splenic CD4^+^ and CD8^+^ cells from PBS‐treated mice were mostly of a Tn phenotype. CD4^+^ T‐cells from IL‐7 treated mice demonstrated an increase in the CD62L_low_ CD44_high_ subset adopting an effector cell‐like phenotype (Figure 3A), whereas CD8^+^ T‐cells were predominantly CD62L_high_ CD44_high_, demonstrating an increase in the Tcm subset (Figure 3C). Interestingly, adding sCD127 significantly enhanced the effect of IL‐7 on T‐cell subsets. CD4^+^ T‐cells demonstrated an increase in Teff cell population from 15% to 22% after IL‐7 treatment, which was increased to 27% after combined treatment (Figure 3A). Similarly, CD8^+^ T‐cells revealed an increase in the Tcm cell population from 17% in PBS treated mice to 47% after IL‐7 treatment and further to 54% after IL‐7 + sCD127 (Figure 3C).

Tn can differentiate into Tcm‐like cells in response to homeostatic proliferation via interaction with self‐antigens and IL‐7 signals.^28^ These differentiated T‐cells are referred to as memory‐like T‐cells. They function and express markers resembling Tcm cells, although they are not generated by contact with a foreign antigen.^29^ In the present study, T‐cell subsets were distinguished by expression of CD62L and CD44.^22^ In healthy mice, IL‐7 treatment resulted in a decrease of CD62L_high_ CD44_low_ CD4 + Tn cells and an increase of CD62L_low_ CD44_high_ CD4 + effector T‐cells in the spleen (Figure 3A). And while IL‐7 also induced a decrease of CD8 + Tn cells (CD62L_high_ CD44_low_), this was associated with an increase of Tcm‐like phenotype (CD62L_high_ CD44_high_) (Figure 3B). Interestingly, the addition of sCD127 significantly enhanced the effect of IL‐7 on the phenotypic changes in CD4^+^ and CD8^+^ T‐cells. This appears to contrast with a previous study where IL‐7 treatment did not induce Tn‐to‐Tcm differentiation in healthy mice.^22^ As the effects of IL‐7 on T‐cells are reversible after treatment cessation (Figure 1C)^30^ it is certainly possible that differentiated T‐cells in our model would revert to pretreatment Tn phenotype.

CD4^+^ and CD8^+^ T‐cell subset numbers in the spleen were next demonstrated (Figure 3B,D). Treatment with IL‐7 significantly increased Tcm and effector T‐cell counts in CD8^+^ T‐cells, whereas CD4^+^ T‐cell subset numbers remained unchanged. With combined treatment both Tcm and effector CD4^+^ and CD8^+^ T‐cell counts were significantly higher when compared with PBS‐treated mice (Figure 3B,D). Of note, there was no noticeable difference in the total naïve CD4^+^ and CD8^+^ T‐cell count between treatment arms. Finally, to determine whether selective cell death might contribute these results, apoptosis of splenic T‐cells was measured by Annexin V staining. Differences in cell viability between treatment arms was trivial and, therefore, unlikely explains the findings above (Figure S3A,B).

## 4 CONCLUDING REMARKS

IL‐7 has potential clinical applications including enhancing immune reconstitution during lymphopenic conditions, for example, cancer patients receiving chemo‐ or radiation therapy. sCD127 significantly enhanced IL‐7‐mediated increases in T‐cell proliferation and number; therefore, IL‐7 + sCD127 treatment is promising for patients with severe T‐cell depletion and may aid T‐cell regeneration following bone marrow transplantation.^31^ In a murine lung cancer model, IL‐7 + sCD127 treatment enhanced antigen presentation, T‐cell activity and inflammation in the tumor microenvironment which reduced tumor burden and improved survival compared with IL‐7 alone.^18^ In conclusion, in healthy mice sCD127 enhances IL‐7‐mediated CD4^+^ and CD8^+^ T‐cell proliferation, increases cell number, and induces Tn differentiation with CD8^+^ T‐cells being more responsive to IL‐7 than CD4^+^ T‐cells. Overall, this study improves the understanding of sCD127 biology, suggests sCD127 can regulate IL‐7 function and suggests benefits for IL‐7 + sCD127 therapy.

## CONFLICT OF INTERESTS

The authors declare that there are no conflict of interests.

## AUTHOR CONTRIBUTIONS

Nawaf A. Aloufi designed and performed the experiments, analyzed all data, and wrote the original draft. Alaa K. Ali helped in performing experiments and data interpretation. Joanne E. McBane helped in writing manuscript and data interpretation, and discussed the results. Stephanie C. Burke Schinkel, Bengisu Molyer, and Priscila O. Barros reviewed and edited the manuscript. Seung‐Hwan Lee and Jonathan B. Angel designed the experiments, supervised the study, helped in data interpretation and discussed the results. Jonathan B. Angel obtained funding.

## Supporting information

Supporting information.

Supporting information.

## Data Availability

Data are available from the corresponding author upon reasonable request.
