# Supplementary figures and images for: Soluble CD127 potentiates IL‐7 activity in vivo in healthy mice
Source: Immun Inflamm Dis. 2021 Sep 15;9(4):1798–808. doi: 10.1002/iid3.530 (PMC8589376; doi:10.1002/iid3.530)

**Suppl  
figure 1**

**A**

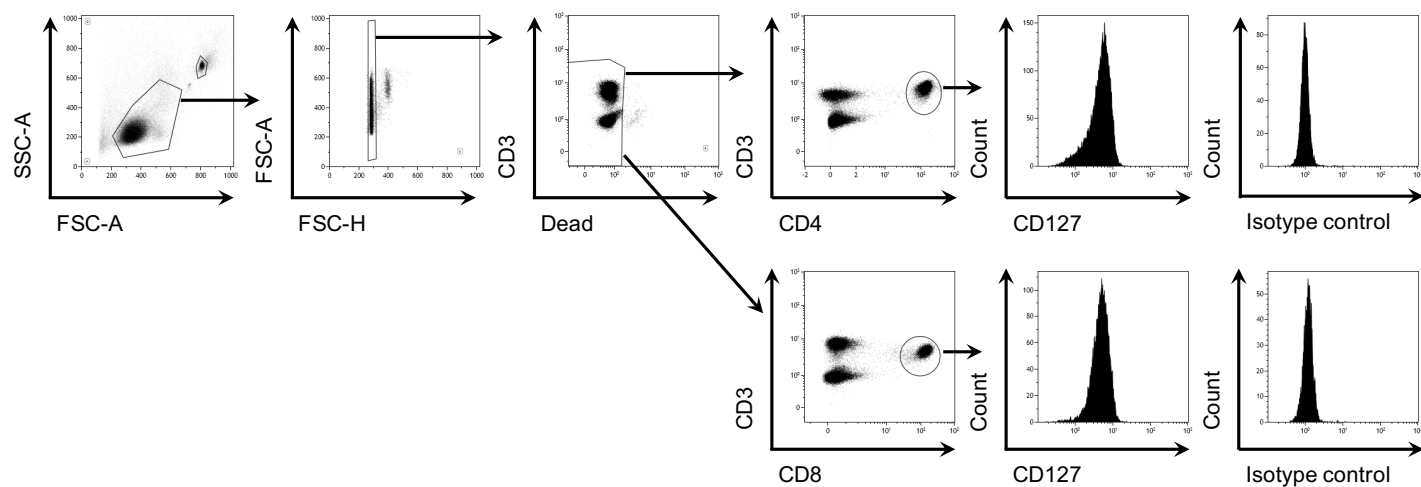

**B**

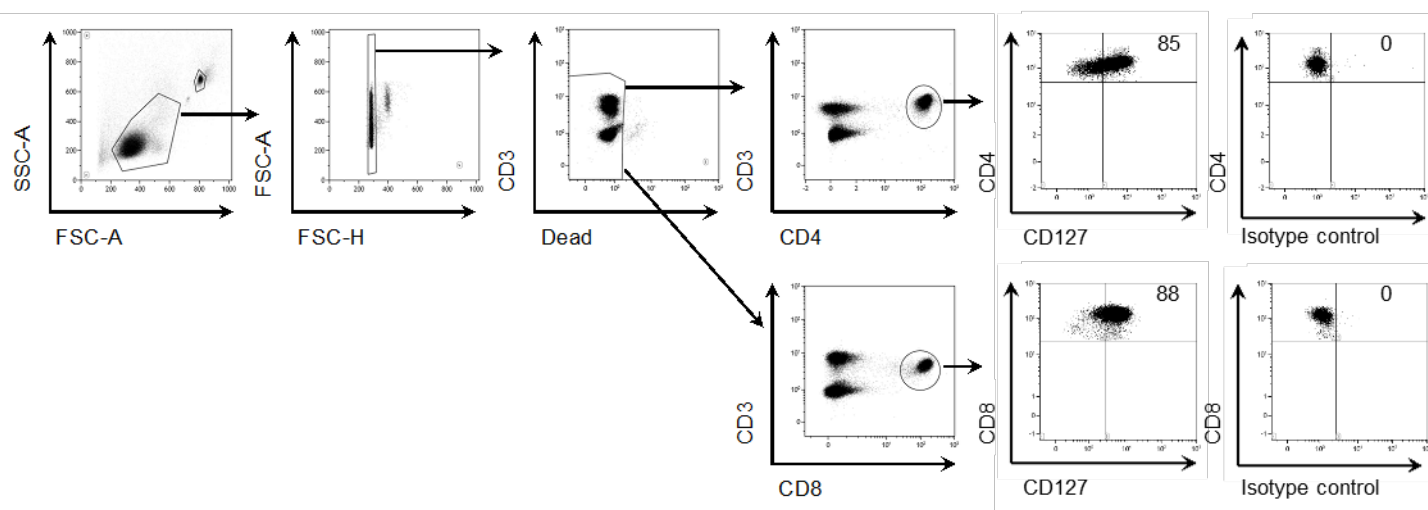

**C**

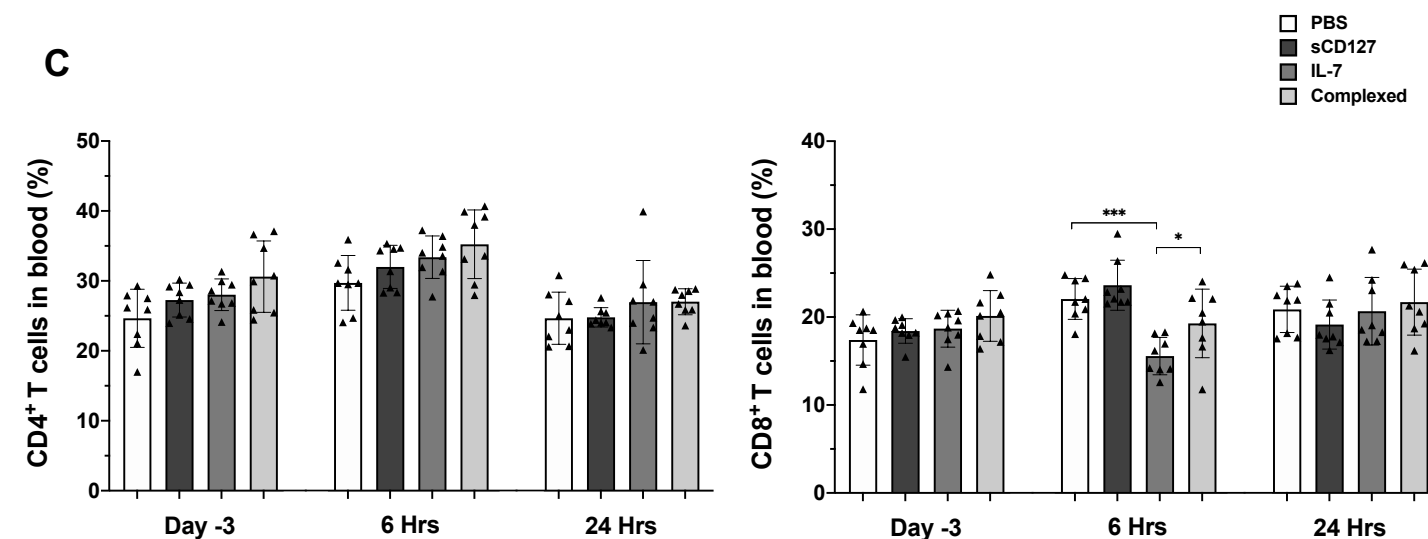

# Suppl figure 2

**A**

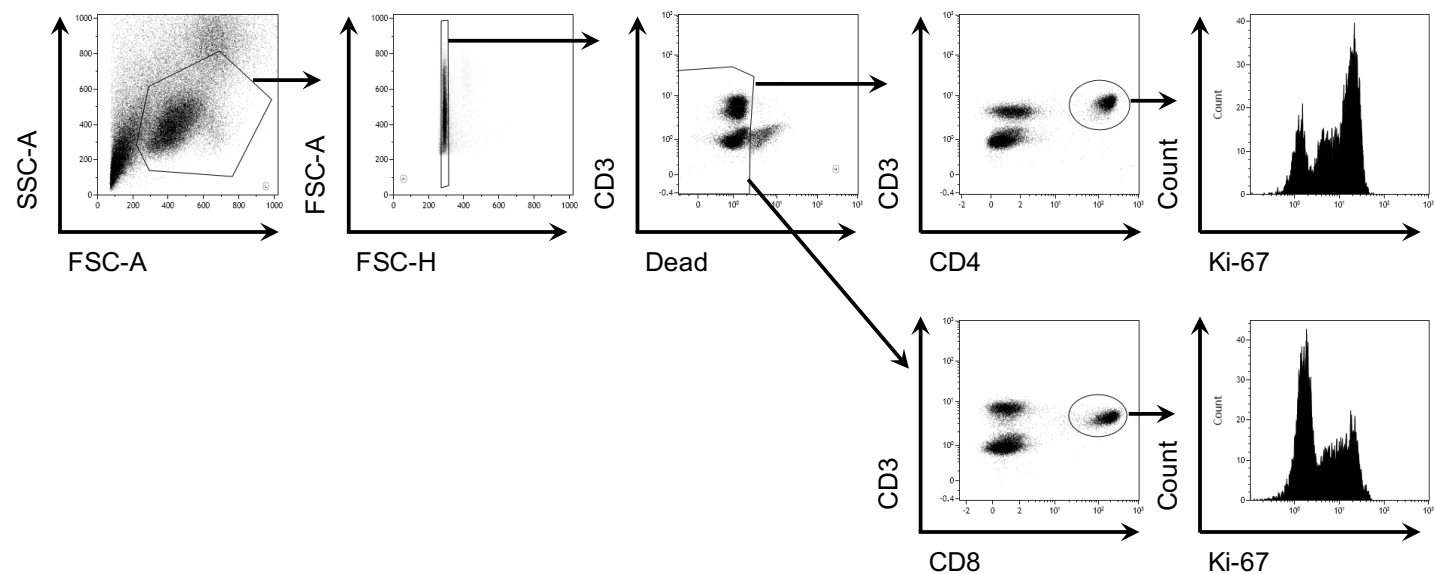

**B**

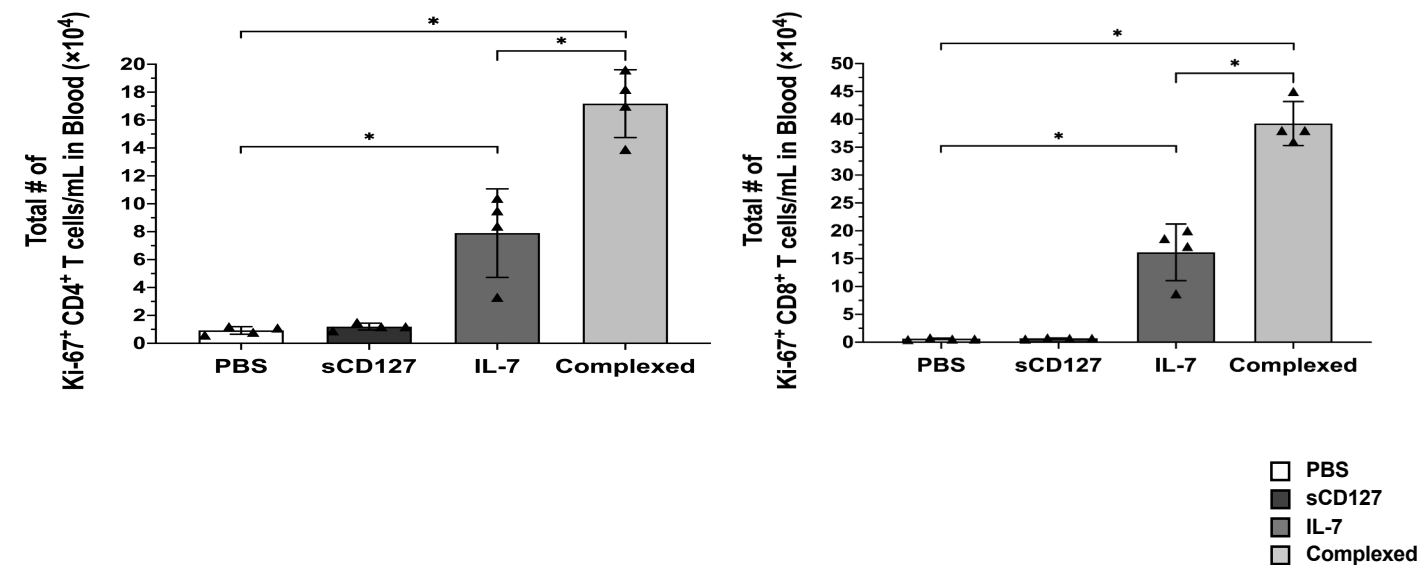

**C**

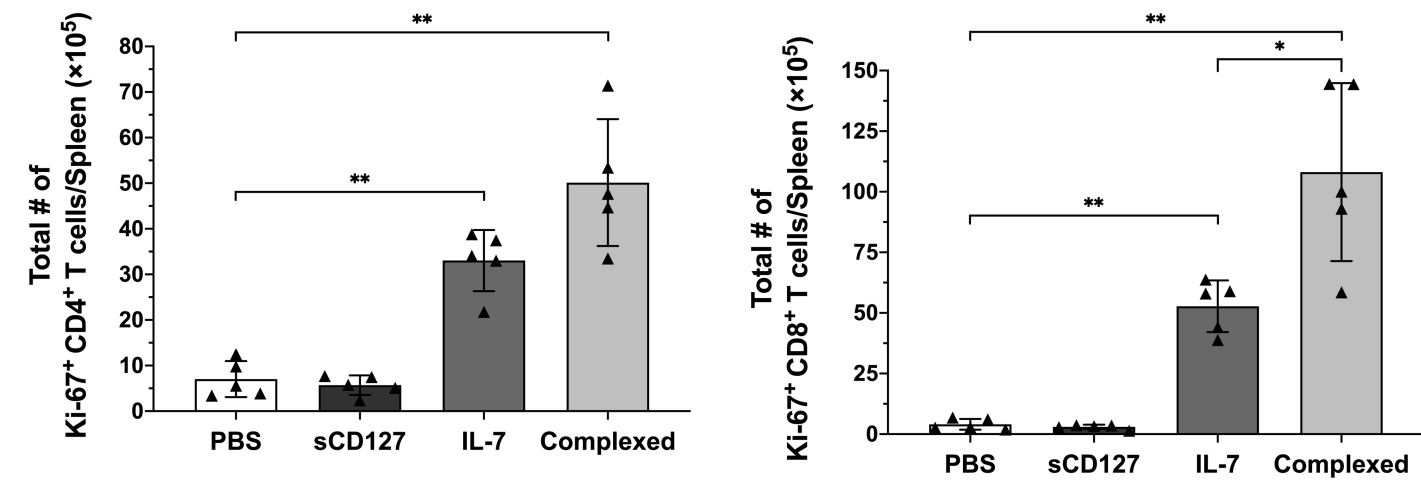

# Suppl figure 3

**A**

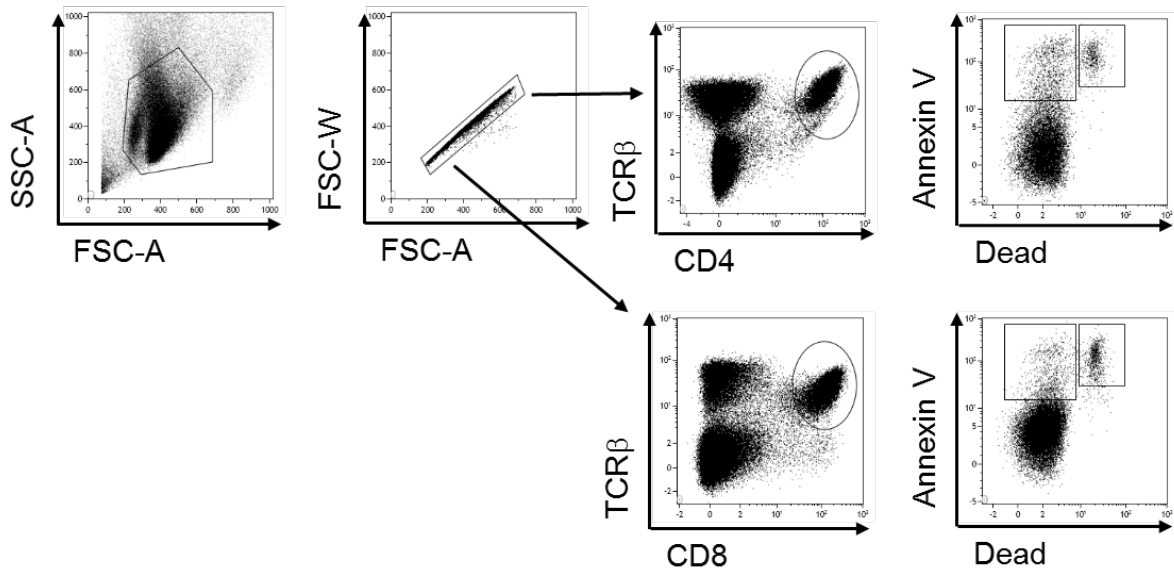

**B**

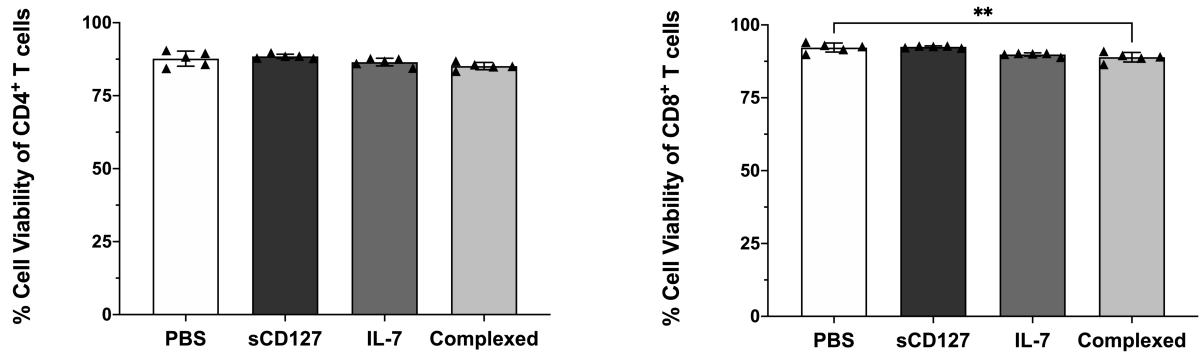

**C**

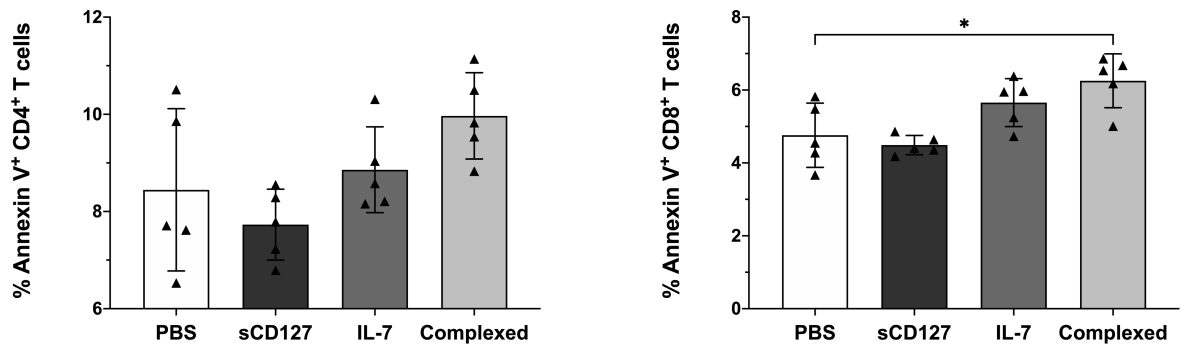

Supplement: Supplementary file 1 — Supporting information. [file IID3-9-1798-s001.pdf]
