# Supplementary material for: Soluble CD127 potentiates IL‐7 activity in vivo in healthy mice
Source: Immun Inflamm Dis. 2021 Sep 15;9(4):1798–808. doi: 10.1002/iid3.530 (PMC8589376; doi:10.1002/iid3.530)
Supplement: Supplementary file 2 — Supporting information. [file IID3-9-1798-s002.docx]

**Soluble CD127 Potentiates IL-7 Activity in vivo in Healthy Mice**

**Authors:** Nawaf A. Aloufi^1, 2, 3^, Alaa K. Ali^1^, Stephanie C. Burke Schinkel^3^, Bengisu Molyer^1, 3^, Priscila O. Barros^3^, Joanne E. McBane^3, 4^, Seung-Hwan Lee^1, 5*^, Jonathan B. Angel^1, 3, 5, 6*^

**Author Affiliation:**

^1^Department of Biochemistry, Microbiology and Immunology, University of Ottawa, Ottawa, Ontario, Canada

^2^King Faisal Specialist Hospital and Research Center, Riyadh, Saudi Arabia

^3^Chronic Diseases Program, Ottawa Hospital Research Institute, Ottawa, Ontario, Canada

^4^Canadian Institutes of Health Research (CIHR) Canadian HIV Trials Network (CTN), Vancouver, British Columbia, Canada

^5^Centre for Infection, Immunity and Inflammation, University of Ottawa, Ottawa, ON, Canada.

^6^Division of Infectious Diseases, Department of Medicine, University of Ottawa and The Ottawa Hospital

^*^**Corresponding Authors:**

Seung-Hwan Lee,

Department of Biochemistry,

Microbiology and Immunology

Faculty of Medicine, University of Ottawa

451 Smyth Road

Ottawa, Ontario, Canada

K1H 8M5

[seunglee@uottawa.ca](mailto:seunglee@uottawa.ca)

Jonathan B. Angel, MD

Division of Infectious Diseases

The Ottawa Hospital

501 Smyth Road, Room G-8

Ottawa, Ontario, Canada

K1H 8L6

Tel: 613-737-8442

Fax: 613-739-6866

[jangel@ohri.ca](mailto:jangel@ohri.ca)

**Keywords:** Interleukin-7 (IL-7), soluble IL-7Rα (sCD127), T cell proliferation. CD4^+^ T cell, CD8^+^ T cell

**Supporting Information**

**Supplementary Figure Legends**

**Figure S1: The effect of IL-7 and sCD127 on CD127 expression on CD4^+^ and CD8^+^ T cells in healthy C57BL/6 mice.** Mice were divided into 4 groups, and treated for 5 consecutive days either with sCD127 alone, IL-7 alone, a combined dose of sCD127 and IL-7 (pre-incubated at 37°C for 30 mins), or PBS. (A) The gating strategy of CD127 analysis from a representative sample. (B) Representative gating strategy for CD127 expressing cells in blood at day -3. (C) The frequency of CD4^+^ T cells **(C left panel)** and CD8^+^ T cells **(C right panel)** in lymphocytes before treatment (Day -3), six hours post treatment (6 Hrs), and twenty-four hours post treatment (24 Hrs). Data are pooled from two independent experiments, n=8 mice per group. Data represent mean ± SD and statistical analysis was performed using an unpaired nonparametric Mann-Whitney test for comparisons between two groups. Significant P values ​​are listed as either *P<0.05, or *** P<0.001.

**Figure S2: The effect of IL-7 and sCD127 on T-cell proliferation and T-cell numbers in the blood and spleen of healthy C57BL/6 mice.** Mice were divided into 4 groups, and treated by i.p. injection for five consecutive days with either: 5 μg of IL-7, 5 μg of sCD127, 10 μg of both treatments complex (pre-incubated for 30 mins at 37°C), or 200 μl of PBS alone (vehicle control). (A) The gating strategy of Ki-67 analysis from a representative sample. (B) Total number of CD4^+^ T cells (B left panel), and CD8^+^T cells (B right panel) expressing Ki-67 in blood are demonstrated. n=4 mice per group. (C) The total number of CD4^+^ T cells (C left panel) and CD8^+^T cells (C right panel) expressing Ki-67 (right) in spleen are shown. n=5 mice per group. Data represent mean ± SD. Statistical analysis was performed using an unpaired nonparametric Mann-Whitney test for comparisons between the two groups. Significant P values ​​are listed as either *P<0.05, or **P<0.01.

**Figure S3: The effect of IL-7 and sCD127 treatment on T-cell viability in the spleen of healthy C57BL/6 mice.** Mice were divided into 4 groups, and treated by i.p. injection for five consecutive days with either: 5 μg of IL-7, 5 μg of sCD127, 10 μg of both treatments complex (pre-incubated for 30 mins at 37°C), or 200 μl of PBS alone (vehicle control). On the day following the final i.p. Injection, mice were sacrificed, and the spleens were harvested. (A) The gating strategy of Annexin V analysis from a representative spleen sample at day 6. (B) Cell viability was assessed using flow cytometry and the determination of Annexin V negative CD4^+^ T cells (B left panel) and CD8^+^ T cells (B right panel). (C) Cell death was assessed using flow cytometry and the determination of Annexin V positive CD4^+^ T cells (C left panel) and CD8^+^ T cells (C right panel). Statistical analysis was completed using an unpaired nonparametric Mann-Whitney test for comparisons between the two groups. Significant P values ​​are listed as either *P<0.05, or **P<0.01.

**Table S1. List of fluorochrome-conjugated antibodies used for flow cytometry**

| **Company** | **Target^a^** | **Reactivity^b^** | **Clone** | **Conjugate^c^** | **Catalogue#** |
| --- | --- | --- | --- | --- | --- |
| BD  Biosciences | CD3 | Ms | 145-2C11 | FITC | 553062 |
|  | CD4 | Ms | RM4-5 | BV786 | 563727 |
|  | CD8α | Ms | 53-6.7 | APC | 553035 |
|  | CD19 | Ms | ID3 | APC/Cy7 and V450 | 557655, 560375 |
|  | CD44 | Ms | IM7 | PE | 553134 |
|  | Ki-67 | H, Ms, Rat, Rh | B56 | V450 and BV421 | 561281, 562899 |
|  | NK1.1 | Ms | PK136 | PE, PE/Cy7, and FITC | 553165, 552878, 553164 |
|  | TCR-β | Ms | H57-597 | FITC | 553171 |
| eBioscience | CD3 | Ms | 145-2C11 | FITC | 11-0031-85 |
|  | CD8α | Ms | 53-6.7 | APC and PE/Cy7 | 17-0081-82, 25-0081-82 |
|  | CD62L | Ms | MEL-14 | FITC | 11-0621-85 |
|  | CD127 | Ms | SB/199 | PE | 12-1273-81 |
|  | TCR-β | Ms | H57-597 | APC and eFluor 450 | 17-5961-83, 48-5961-82 |
| BioLegend | CD44 isotype | Rat | RTK4530 | PE | 400608 |
|  | CD62L isotype | Rat | RTK2758 | FITC | 400506 |
|  | CD127 isotype | Rat | RTK4530 | PE | 400608 |

^a^Target abbreviations: CD-Cluster of differentiation; CD62L-CD62 ligand; NK1.1 – Natural killer cell marker/antigen 1.1; TCR-beta – T cell receptor-beta.

^b^Reactivity abbreviations: Ms-mouse; H-human; Rh-Rhesus monkey; Sh-sheep.

^c^Conjugate abbreviations: APC-Allophycocyanin, FITC-fluorescein isothiocyanate, PE-phycoerythrin; PE/Cy7-phycoerythrin/Cyanine 7.
